# Identification and hazard prediction of tattoo pigments by means of pyrolysis—gas chromatography/mass spectrometry

**DOI:** 10.1007/s00204-016-1739-2

**Published:** 2016-05-21

**Authors:** Ines Schreiver, Christoph Hutzler, Sarah Andree, Peter Laux, Andreas Luch

**Affiliations:** Department of Chemical and Product Safety, German Federal Institute for Risk Assessment (BfR), Berlin, Germany

**Keywords:** Tattoo inks, Pyrolysis, Organic pigments, Tattoo toxicity, Pigment degradation

## Abstract

The implementation of regulation for tattoo ink ingredients across Europe has generated the need for analytical methods suitable to identify prohibited compounds. Common challenges of this subject are the poor solubility and the lack of volatility for most pigments and polymers applied in tattoo inks. Here, we present pyrolysis coupled to online gas chromatography and electron impact ionization mass spectrometry (py-GC/MS) as quick and reliable tool for pigment identification using both purified pigments and tattoo ink formulations. Some 36 organic pigments frequently used in tattoo inks were subjected to py-GC/MS with the aim to establish a pyrogram library. To cross-validate pigment identification, 28 commercially available tattoo inks as well as 18 self-made pigment mixtures were analyzed. Pyrograms of inks and mixtures were evaluated by two different means to work out the most reliable and fastest strategy for an otherwise rather time-consuming data review. Using this approach, the declaration of tattoo pigments currently used on the market could be verified. The pyrolysis library presented here is also assumed suitable to predict decomposition patterns of pigments when affected by other degradation scenarios, such as sunlight exposure or laser irradiation. Thus, the consumers’ risk associated with the exposure to toxicologically relevant substances that originate from pigment decomposition in the dermal layers of the skin can be assessed. Differentiation between more or less harmful pigments for this field of application now will become feasible.

**Electronic supplementary material:**

The online version of this article (doi:10.1007/s00204-016-1739-2) contains supplementary material, which is available to authorized users.

## Introduction

Over the past few decades, inorganic pigments as coloring agents in tattoo inks were succeeded by organic compounds. This development was mainly triggered by an increased color brilliance of the latter and their lower contamination with heavy metal impurities such as nickel, chromium or cobalt (Dirks 2015). In addition, the European Resolution on requirements and criteria for the safety of tattoos and permanent makeup [ResAP(2008)1] highlighted pigments that should not be used in tattoo inks. This judgement was based on the EU cosmetics regulation (Council of Europe 2008). In the past years, ResAP(2008)1 was converted into law by some countries, including Germany, thereby prohibiting some 100 colorants (TätoV 2008). However, the implementation of this regulation can only succeed if pigments become accessible by analytical means used in routine monitoring. Previous studies have reported up to two-third of false declaration of tattoo inks with one-third of the inks containing prohibited pigments (Hauri and Hohl 2015). The lack of standard methods for the identification of pigments in tattoo inks leaves space to bypass regulation simply by non-detectable false declaration.

To date, identification of tattoo pigments is mainly carried out using liquid chromatography (LC) in the case of soluble pigments (Engel et al. 2006) or, if insoluble, Raman spectroscopy as well as Fourier transform infrared (FT-IR) spectroscopy (Poon et al. 2008; Timko et al. 2004). In addition, techniques of inorganic element analysis such as inductively coupled plasma mass spectrometry (ICP-MS) (Forte et al. 2009) and energy dispersive X-ray spectroscopy (EDX) are applied to characterize tattoo inks (Taylor et al. 1991; Timko et al. 2004). Recent investigations proved laser desorption ionization time-of-flight mass spectrometry (LDI-ToF–MS) also being suitable for pigment identification (Boon and Learner 2002; Hauri 2014). Disadvantages of single methods are either their low sensitivity and/or their specificity toward organic pigment mixtures (FT-IR and Raman spectroscopy), an insufficient solubility of the analytes under assay conditions (LC–MS), rather long data evaluation times (LDI-ToF–MS) and the lack of suitable spectra libraries (LDI-ToF–MS). Therefore, combinations of complementary methods such as LC and LDI-ToF–MS have been used in the past to identify coloring agents in tattoo inks (Hauri 2011, 2014).

Organic pigments in paintings and varnishes are often identified using pyrolysis coupled to gas chromatography/mass spectrometry (py-GC/MS) (Chiantore et al. 2003; Ghelardi et al. 2014; Russell et al. 2011; Sonoda 1999). Pigments are first subjected to decomposition at high temperatures, and emerging pyrolysis product patterns are then used to identify the corresponding parent compounds. With this method, polymers can be simultaneously analyzed as well (Chiantore et al. 2003; Kleinert and Weschler 1980; Schossler et al. 2013; Silva et al. 2010; Wallisch 1974). In the literature, the specific patterns of main pigment fragments have been reported, unfortunately sometimes lacking unidentified fragments or smaller molecules with toxicological relevance (Chiantore et al. 2003; Ghelardi et al. 2014; Russell et al. 2011; Sonoda 1999). Losing some unique fragments makes identification of pigments more challenging though. Additionally, applied pyrolysis temperatures greatly vary between publications. Therefore, we tested the influence of different temperatures on the thermal stability of various pigments. Ultimately we provide a full database of the main fragments emerging upon pyrolysis of 36 among the most widely used organic tattoo pigments at 800 °C. The feasibility of this pyrogram library was then tested on 28 commercially available tattoo inks from different German and international manufacturers. Additionally, 18 self-made pigment mixtures were used to appraise limitations of the method.

The pyrolysis products presented in this paper were also reviewed for their toxicological properties. Recent studies have provided evidence that products of pyrolysis will be comparable to those obtained by laser irradiation (Engel et al. 2007; Schreiver et al. 2015), and some of them were also shown to occur upon sunlight exposure of pigments in vitro as well as in vivo (Cui et al. 2004; Engel et al. 2007, 2010; Hauri and Hohl 2015; Wenzel et al. 2013; Wezel 2013). Hence, the compiled pyrolysis fragments might be suitable to predict hazardous decomposition products of organic pigments that possibly evolve during laser irradiation or sunlight exposure of tattooed skin.

## Materials and methods

### Chemicals

For verification of retention times and mass spectra, chemical substances were either purchased from Sigma-Aldrich (St Louis, MO, USA) with purities of ≥97.0 % (sodium cyanide, benzonitrile, 1,2-benzenedicarbonitrile, chlorobenzene, 4-chlorobenzonitrile, xylene, benzamide, 2-ethoxyaniline) or, as analytical standards, from Fluka (Sigma-Aldrich, St Louis, MO, USA; naphthalene, 1,2,3,4-tetrachlorobenzene, hexachlorobenzene, pentachlorobenzene, aniline, 3,3′-dichloro-1,1-biphenyl, 2,5-dichloroaniline) and Sulpeco (i.e., Sigma-Aldrich; pentachlorobenzene). Shellac was obtained as food-grade and orange shellac from Kremer Pigmente (Aichstetten, Germany).

### Py-GC/MS

A 7890A gas chromatograph system coupled to a 5975C inert XL MSD with Triple-Axis Detectors (both from Agilent Technologies, Waldbronn, Germany) was used. Ionization was induced with an inert electron impact (EI) ion source at 70 eV and helium (purity of 99.999 %) from Air Liquide (Düsseldorf, Germany) was used as carrier gas.

For py-GC/MS the gas chromatograph was equipped with an HP-5MS column (30 m × 0.25 mm i.d. × 0.25 µm; Agilent Technologies, Waldbronn, Germany). Small samples of pigments or tattoo inks were placed inside a glass tube and then automatically inserted into the pyrolysis module of the thermal desorption unit (TDU) (both from Gerstel, Mühlheim, Germany) of the GC/MS inlet system. For tattoo inks, a solvent vent method was used to dry the samples and to analyze for semi-volatile compounds prior to the onset of pyrolysis. Therefore, the TDU was ramped after 0.5 min from initial 50–90 °C (100 °C/min) and kept at this temperature for further 1.5 min. The solvent vent quit after 1.9 min. Afterward, the TDU was heated to 320 °C (720 °C/min) for 3.5 min to evaporate volatile compounds, which were subsequently captured in the Cold Injection System (CIS) at −150 °C. The temperature of the TDU was held for 2 min before ramping to 320 °C (12 °C/min). The starting temperature of the oven was held at 50 °C for 2 min and then ramped with 10 °C/min to reach 320 °C for 5 min. The carrier gas flow rate was 1 ml/min with a split ratio of 1:30.

Pyrolysis of dried inks and pigments was carried out at varying temperatures for 6 s. Parameters for the pyrolysis of pure pigments were the same as stated above with following adaptations: The temperature of the CIS was kept constant at 320 °C. The TDU was ramped from 50 to 320 °C (720 °C/min) and then kept constant for further 1.6 min.

Data were analyzed using Enhanced ChemStation (E02.02.1431) from Agilent Technologies. Firstly, the mass spectra recorded were compared to the mass spectral library of unknown peaks (see Tables S1–11) considering all peaks with a peak area of ≥0.2 % of the total. If matches were below 80 with the self-made library (ChemStation score, with 100 being the best possible match), the NIST MS library (MS Search version 2.0 g) was used for spectral comparison. In this case, matches with scores higher than 90 were accounted true.

Altogether 36 pigments were pyrolyzed at 800 °C to identify specific cleavage sites of each pigment (Table S12). If a pigment was in stock from more than one supplier, only mutual peaks of the respective pyrograms were depicted in Tables S1–11.

### Pigment mixtures

For suspension of pigments, 18 g glycerol and 15 g propylene glycol (≥99.5 %) were dissolved in a 40/60 (v/v) mix of 2-propanol (≥99.5 %, all from Sigma-Aldrich, St Louis, MO, USA) and deionized water (MilliQ Advantage A10; Merck, Darmstadt, Germany) with a resistance of 18.2 MΩ at 25 °C. 50 mg of each pigment was added to 3 ml of the suspension. Homogenization took place in a Sonorex Digitec ultrasonic water bath with 50/60 Hz (Bandelin Electronic, Berlin, Germany) for 60 min at <40 °C.

## Results and discussion

Thirty-six pigments have been pyrolyzed at 800 °C to create a library of specific pyrolysis decomposition patterns (Tables S1–11). The pigments investigated were chosen according to their popularity in use for tattoo inks taken from ink declarations and published surveys (BAG 2009; CVUA 2011).

**Fig. 1:**
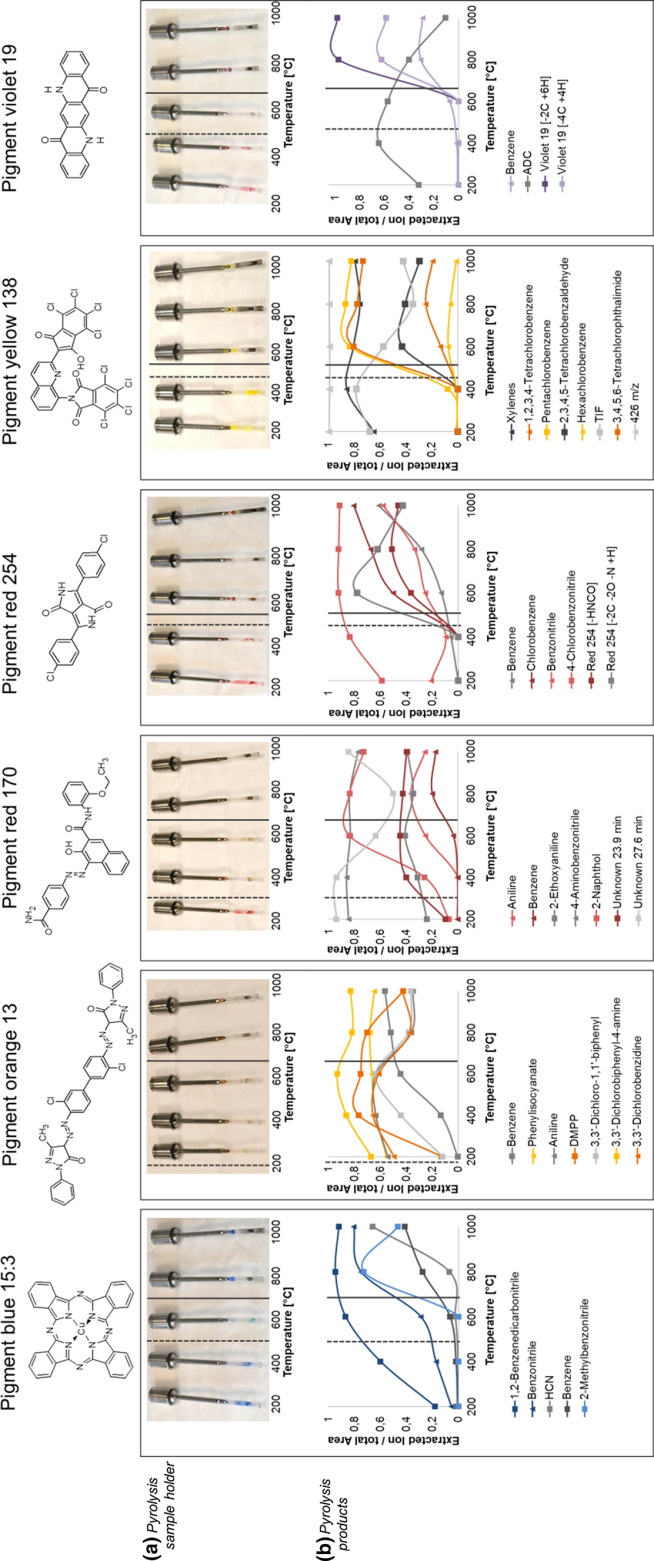
Temperature-dependent pyrolysis of six common tattoo pigments. Degradation of pigments is indicated by carbonization of the test tubes (**a**) and the increase in cleavage products (**b**). The onsets of decoloration and carbonization were indicated with *dashed* and *solid lines*, respectively. **b** Non-quantitative increases in pyrolysis products are displayed as extracted molecular mass of each fragment normalized to the total chromatogram area. 426 m/z, 2-(8-aminoquinolin-2-yl)-4,5,6,7-tetrachloro-3-hydroxy-1*H*-inden-1-one; ADC, 2-amino-9-oxo-dihydroacridine-3-carbaldehyde; DMPP, 2,4-dihydro-5-methyl-2-phenyl-3*H*-pyrazol-3-one; HCN, hydrogen cyanide; TIF, 4,5,6,7-tetrachloro-1,3-isobenzofuradione

To determine a suitable pyrolysis temperature, six pigments which cover the most abundant organic structures used in tattoo inks were pyrolyzed at 200, 400, 600, 800 and 1000 °C. Areas of the extracted molecular mass ions, normalized to the total chromatogram area, were found increasing and thus confirm the expected temperature-dependent formation of decomposition products (Fig. 1). Some pigments, such as pigment orange (P.O.) 13 and pigment red (P.R.) 170, decompose at rather low temperatures (<400 °C) which becomes apparent by rising peak ratios of cleavage products and color changes in sample holders (Fig. 1). In these cases, the thermal instability is caused by incorporated azo bonds which are prone to cleavage already at temperatures starting at 200 °C (Az et al. 1991). On the other hand, extremely stable pigments such as pigment violet (P.V.) 19 and pigment blue (P.B.) 15 remain more or less unaffected below 800 °C. Based on these observations, a pyrolysis temperature of 800 °C was chosen for the generation of a pyrogram library and the following tattoo ink analyses to ensure cleavage of all targeted pigments.

Occasionally it was impossible to identify all pyrolysis products using either an MS library, as provided by the US National Institute of Standards and Technology (NIST), or via judgement of the mass spectrum taking into account the pigment’s molecular structure. Since the occurrence of such as yet unknown molecule descendants is unique to certain pigments, we added these fragments as unknowns to the lists of decomposition products. Basically all pyrolysis products representing >1 % of the total peak area were included in the pyrogram library, with a few exceptions only (Tables S1–11). In the following, all different classes of organic pigments used in tattoo inks are discussed in terms of their main pyrolysis products and accompanying toxicological hazards.

### Phthalocyanines

1,2-Benzenedicarbonitrile and its halogenated derivatives are the most abundant products emerging upon pyrolysis of the phthalocyanines P.B.15, pigment green (P.G.) 7 and P.G.36, respectively (Table S1). Because these molecules do not occur in the pyrograms of any other pigment analyzed, they can be used for the identification of phthalocyanines. In addition, benzonitrile and phthalimide result from pyrolysis of P.B.15. Since the latter compound is used in the synthesis of the pigment, its presence likely indicates incomplete purification. Highly toxic cyanide compounds including hydrogen cyanide, cyanogen chloride and cyanogen bromide are of special concern among all pyrolysis products of phthalocyanines. Recently we have demonstrated that hydrogen cyanide is also released upon ruby laser irradiation of P.B.15 (Schreiver et al. 2015).

Copper-containing phthalocyanines are the only blue- and green-colored organic pigments present in tattoo inks so far (BAG 2009; CVUA 2011). However, P.B.15 and P.G.7 are listed in annex 1 of the cosmetics regulation in Germany whose substances are forbidden for usage in tattoo inks according to the German law (TätoV 2008). Since the comment “when used as a substance in hair dye products” was added to both pigments, their actual legal status concerning an application in tattoo inks is not always interpreted in the same way. Nonetheless, P.G.7 was listed as forbidden pigment in a governmental market survey (CVUA 2011).

### Azo pigments

For identification of azo pigments, cleavage of azo and amide bonds of specific residues attached to the core structures such as naphthols or biphenyls result in the appearance of characteristic fragments (Tables S2–6). Since similar coupling groups are used in many different pigments, identification depends on the occurrence of specific fragmentation patterns. For small pigments such as P.R.4 and P.O.5, even the unfragmented molecule ion can enter the gas phase and thus can be easily detected via py-GC/MS (Table S2). To our knowledge, P.O.34, which was found in tattoo inks lately, has not been described in pyrolysis studies before (Table S3) (BAG 2009; CVUA 2011). Similar to other diazo pigments, P.O.34 is cleaved at the azo bonds, thereby releasing the carcinogenic primary aromatic amine (pAA) 3,3′-dichlorobenzidine. Other carcinogenic pAAs such as aniline, *o*-anisidine or *o*-toluidine originate from the cleavage of coupling groups of azo and diazo pigments, too. In the past, the release of carcinogenic pAAs upon sunlight exposure and laser irradiation, along with the occurrence of allergic reactions mainly reported with red and yellow tattoos that presumably contained azo pigments, shed bad light on their usage (Cui et al. 2004; Engel et al. 2007; Gaudron et al. 2015; Hauri and Hohl 2015; Vasold et al. 2004; Wezel 2013).

### Diketopyrrolopyrroles **(**pyrrolo[3,2-*b*]pyrrole-diones)

Most abundant fragments of both P.O.73 and P.R.254 pigments are benzonitriles with either *tert*-butyl or chlorine residues depending on the respective parent compound (Table S7). In these cases, cleavage occurs inside the ketopyrrol rings as indicated. In recent years, diketopyrrolopyrroles were preferentially used by German tattoo ink manufacturers to replace the disputed azo pigments (Hauri 2011). Just as by any other organic pigment, carcinogens like benzene or, in the case of P.O.73, naphthalene are formed at temperatures of 800 °C and higher.

### Quinophthalones (2-(2-quinolyl)-1,3-indandiones)

The pyrogram of pigment yellow (P.Y.) 138 (3,4,5,6-tetrachloro-*N*-[2-(4,5,6,7-tetrachloro-2,3-dihydro-1,3-dioxo-1*H*-inden-2-yl)-8-quinolyl]phthalimide) contained a characteristic molecule with an m/z ratio of 426 and some smaller peaks of different chlorinated benzenes. Among them, the human category 1B carcinogens hexachlorobenzene and 4,5,6,7-tetrachloro-1,3-isobenzofurandione (TIF) can be found (Table S8). The relative peak areas of TIF, the unknown 426 m/z molecule and xylene were not increasing at decay temperatures of 600 °C and higher (Fig. 1). In the case of TIF, the molecule abundance even strongly decreased at higher temperatures. It is thus likely that all three molecule species represent impurities degradable only at higher temperatures. The quinophthalone P.Y.138 is especially used in tattoo inks manufactured in Germany. To our knowledge, data on the pyrolysis of this pigment have never been presented before.

### Quinacridones (5,12-dihydroquino[2,3-*b*]acridine-7,14-diones)

Due to their high stability (i.e., lack of weak bonds), temperatures above 600 °C are needed to induce pyrolysis of quinacridones (Fig. 1, Table S9). Cleavage occurs at the indicated sites within the pyridone rings, thereby forming benzenes with pigment-specific residues attached (Table S9). Besides that, detected fragments were due to the loss of carbons and hydrogens or resulted from the rearrangement of the parent compound leading to molecule species that could not be further characterized. Absolute peak areas of quinacridone pyrolysis products were relatively low, thus making high amounts of pigments necessary for py-GC/MS analysis. Since pigment concentrations in tattoo ink mixtures were too low quinacridone identification via py-GC/MS remained unfeasible.

The only known toxic compound evolving from pyrolysis of quinacridones is the human carcinogen benzene. On the other side, as yet unknown fragments emerging during pyrolysis leave an uncertainty in the toxicological assessment of these pigments.

Unlike chlorinated P.R.202, the pigments P.R.122 and P.V.19 are listed in annex I of the cosmetics regulation with the addition “when used as a substance in hair dye products”; thus these pigments are accounted as being prohibited for usage in tattoo inks in Germany as well (CVUA 2011). Nonetheless, all three pigments are still frequently used to create magenta to violet color shades due to their high color brilliance.

### Triphendioxazines (“dioxazines”)

P.V.23 is mainly cleaved into the class 2 carcinogen 9*H*-carbazole and a highly abundant unknown product with an m/z ratio of 211 (Table S10). P.V.37 is cleaved into a variety of molecules due to the multitude of weak bonds in its structure. Among pyrolysis-induced degradation products the carcinogen benzene emerged at high levels with >8 % of the total peak area (Table S10).

In contrast to P.V.37, P.V.23 was banned from use in tattoo inks by German legislation. Nonetheless, both pigments can be found in tattoo inks sold elsewhere in Europe and thus need to be monitored and analytically separated from each other (Hauri 2011). Pyrolysis of both triphendioxazine pigments has previously been shown by Ghelardi et al. (2014).

### Other polycyclic organic pigments

The replacement of azo pigments led to the introduction of a variety of chemical classes as coloring agents, namely perinone (P.O.43), anthraquinones (e.g., P.R.177), perylenes (e.g., P.R.179) and rhodamines (e.g., rhodamine B) (Table S11). Due to their compact polycyclic structure, P.O.43 and P.R.179 give rise to rather unspecific decomposition products, whereas P.R.177 can be easily identified through the occurrence of 1-amino-9,10-anthracenedione. Rhodamine B is cleaved at both its amine moiety and at the carboxyl group to yield a variety of different pyrolysis products. Benzene is the only known carcinogen formed from these polycyclic organic pigments at high pyrolysis temperatures.

P.O.43 is prohibited for use in tattoo inks, whereas P.R.177 and P.R.179 are not regulated. Rhodamine B (C.I. 45170) and its hydroxide form (45170:1) are forbidden in accordance to the German tattoo regulation (TätoV 2008). It still can be found in tattoo inks though (Hauri 2011).

### Polymers and additives

Besides pigments, common polymers and thickeners used in tattoo inks have also been analyzed. Polymers decompose into their primary building blocks and other secondary pyrolysis products (Table 1). Some primary structures such as the carcinogen *N*-vinylpyrrolidone are of major concern. Residues of this monomer remaining in the polymer polyvinylpyrrolidone (PVP) upon synthesis or emerging during metabolism or degradation of PVP are thus to be excluded (Klimisch et al. 1997). The polymer PVP as such is non-toxic; however, when administered in large amounts and at molecular weights above 20,000, it might lead to localized cutaneous PVP storage disease (Chi et al. 2006).

**Table 1:** Pyrolysis products of polymers

Name	Fragments	m/z	Toxicology (GHS)^a^
Silicones	Dimethylcyclosiloxanes (oligomers)	207 (D3)	–
		281 (D4)	Reproductive toxicity, Cat. 2
		370 (D5)	–
Polyethylene glycol	Ethylene glycol (oligomers)	133, 89, 45, (common masses of oligomers)	Specific target organ toxicity, repeated exposure (kidney), Cat. 2
Polyvinylpyrrolidone	Benzene	78	Carcinogenicity, Cat. 1A
	1-Methyl-2-pyrrolidinone	99	Skin irritation, Cat. 2
	*N*-Vinylpyrrolidone	111	Carcinogenicity, Cat. 2
Acrylates	Methyl methacrylate	100	Skin sensitizer, Cat. 1
	Ethyl methacrylate	114	Skin sensitizer, Cat. 1
	Dodecyl methacrylate	254	Skin irritation, Cat. 2
	Tetradecyl methacrylate	282	n.a.
Polystyrene	Styrene	104	Skin irritation, Cat. 2
			Specific target organ toxicity, repeated exposure, Cat. 1
	α-Methylstyrene	118	Eye irritation, Cat. 2
Shellac	Benzene	78	Carcinogenicity, Cat. 1A
	Toluene	91	Skin irritation, Cat. 2
			Reproductive toxicity, Cat. 2
	Styrene	104	Skin irritation, Cat. 2
			Specific target organ toxicity, repeated exposure, Cat. 1
	Naphthalene	128	Carcinogenicity, Cat. 2
	Biphenyl	154	Skin irritation, Cat. 2

Fragments of the six polymers listed were found in the pyrograms of tattoo inks. With the exception of acrylates, authentic standards of all polymers were pyrolyzed to verify specific decomposition products. Toxicity was listed according to GHS classification (IFA 2016)

^a^Carcinogenicity, Cat. 1A: Known to have carcinogenic potential in humans (evidence from human epidemiology); Carcinogenicity, Cat. 2: Suspected human carcinogen; Eye irritation, Cat. 2: Reversible eye effects; Reproductive toxicity, Cat. 2: Suspected human reproductive or developmental toxicant; Skin irritation, Cat. 2: Irritant; Skin sensitizer, Cat. 1: Evidence in humans that the substance can lead to sensitization by skin contact in a substantial number of persons or positive results from an appropriate animal test; Specific target organ toxicity, repeated exposure, Cat. 1: Substances that have produced significant toxicity in humans, or that, on the basis of evidence from studies in experimental animals can be presumed to have the potential to produce significant toxicity in humans following repeated exposure; Specific target organ toxicity, repeated exposure, Cat. 2: Substances that, on the basis of evidence from studies in experimental animals, can be presumed to have the potential to be harmful to human health following repeated exposure

Pyrolysis of silicones (polydimethylsiloxanes) resulted in the formation of cyclic dimethylsiloxanes (Table 1). Silicones, but also different dimethylcyclosiloxanes are used for suspension and as anti-foaming agents. However, py-GC/MS analytics cannot distinguish between linear and cyclic siloxanes such as D4 (octamethylcyclotetrasiloxane) and D5 (decamethylcyclopentasiloxane), respectively. While cyclic D4 siloxanes have revealed only low estrogenic activity, D5 siloxanes increased the rate of uterine tumors in animal studies (OEHHA 2008). Also, D5 is presumably interfering with the neurotransmitter dopamine and the hormone prolactin. Based on these data the use of silicones should be further evaluated in terms of tattoo regulation.

Polyethylene glycol (PEG) can be metabolized into low molecular weight oligomers and its hydroxy acid and diacid derivatives, or even toward the monomer ethylene glycol (MAK 1998). This metabolic degradation pattern of PEG could be recapitulated through py-GC/MS (Table 1). The hydrophilic metabolites of PEG will be excreted via urine but can also trigger acidosis at high concentrations due to an increased serum osmolarity and the formation of calcium complexes. Ultimately this may lead to renal and heart failure (MAK 1998; Parry and Wallach 1974).

Since styrene was found after pyrolysis of tattoo inks (data not shown), its generation has been verified via pyrolysis of polystyrene (Table 1). Pyrolysis of polystyrene indeed resulted in the formation of styrene, α-methylstyrene, styrene dimers and higher building blocks. Among these degradation products, styrene has been shown to be metabolized into styrene 7,8-oxide, an intermediate categorized as carcinogen 1B according to GHS classification that can also trigger contact allergy (Ohtsuji and Ikeda 1971; Sjöborg et al. 1894). Polystyrene can be used in pigment synthesis to facilitate particle distribution in aqueous dispersions, an application explaining its occurrence in tattoo inks (Tsubokawa et al. 1999).

Some manufacturers also use natural thickeners such as shellac. Shellac is an organic resin which only fragmented into unspecific products such as benzene, toluene and naphthalene during pyrolysis (Table 1). Interestingly, also styrene was formed upon pyrolysis of shellac, but not α-methylstyrene which only appeared in polystyrene pyrolysis (Table 1).

### Pyrolysis of tattoo inks

In total, we looked into the pyrolysis-mediated degradation of 28 tattoo inks which were, according to their labeling, supposed to contain pigments that have been included in our pyrogram library (Table 2). Additionally, 18 self-made mixtures along with some rather “challenging” pigments were pyrolyzed.

**Table 2:** Identification of pigments in tattoo inks and self-made mixtures

No.	Tattoo inks—organic pigments declared at the label	Identified by average mass spectrum (AMS)	Identified by fragment comparison
1	None, blue color	**–**	P.B.15
2	P.B.15	P.B.15	P.B.15
3	P.B.15	P.B.15	P.B.15
4	P.B.15	P.B.15	P.B.15
5	**P.B.15**, P.R.170	P.R.170 (or P.R.210)	P.R.170 (or P.R.210)
6	P.B.15, **P.R.202**, **P.V.37**	P.V.37	P.B.15
7	P.G.36	P.G.36	P.G.36
8	P.G.36, P.Y.154	P.Y.154	P.G.36, P.Y.154
9	**P.O.13**, P.Y.65	P.Y.74 (or P.Y.65)	P.Y.74 (or P.Y.65)
10	**P.O.13**, P.R.210	P.R.170 (or P.R.210)	*P.R.146*, P.R.170 (or P.R.210)
11	P.O.16, **P.Y.14**	P.O.16	*P.Y.1*, P.O.16
12	P.O.73, P.Y.138	P.O.73	P.O.73, P.Y.138
13	P.O.73, P.Y.138	P.Y.138	P.O.73, P.Y.138
14	**P.O.73**, P.Y.97, **P.R.202**	P.Y.97	P.Y.97
15	P.R.170	P.R.170 (or P.R.210)	P.R.170 (or P.R.210)
16	P.R.170	P.R.170 (or P.R.210)	P.R.170 (or P.R.210)
17	P.R.177	P.R.177	P.R.177
18	P.R.254	P.R.254	P.R.254
19	P.R.254	P.R.254	P.R.254
20	P.R.254	P.R.254	P.R.254
21	P.R.254, P.R.177	P.R.254	P.R.177, P.R.254
22	P.Y.14	P.Y.14	*P.Y.74*, P.Y.14
23	P.Y.14	P.Y.14	P.Y.14
24	P.Y.65	P.Y.74 (or P.Y.65)	P.Y.74 (or P.Y.65)
25	P.Y.138	*P.B.15*	P.Y.138
26	P.Y.138	P.Y.138	P.Y.138
27	P.Y.138	P.Y.138	P.Y.138
28	P.Y.154	P.Y.154	P.Y.154

Sum	Different from declaration Missing	2/28 inks = 7.1 % Not applicable	3/40 pigments = 7.5 % 8/40 pigments = 20 %
Mix 1	P.B.15, P.V.23	*P.R.254*	P.B.15, P.V.23
Mix 2	**P.O.43**, P.R.112	P.R.112	P.R.112
Mix 3	P.O.73, P.R.254	P.R.254	P.O.73, P.R.254
Mix 4	P.O.5, P.Y.83	P.Y.83	P.O.5,P.Y.83
Mix 5	P.O.16, P.Y.14	P.Y.14	P.O.16, P.Y.14
Mix 6	P.R.112, **P.R.202**, P.R.254	*P.Y.138*	P.R.112, P.R.254
Mix 7	P.R.4, P.Y.3	P.R.4	P.R.4, P.Y.3
Mix 8	P.R.5, P.Y.83	P.Y.83	P.R.5, P.Y.83
Mix 9	P.R.5, P.Y.97	P.Y.97	P.R.5, P.Y.97
Mix 10	P.R.5, P.Y.1	P.R.5	P.R.5, P.Y.1
Mix 11	P.R.22, P.Y.1	P.Y.1	P.R.22, P.Y.1
Mix 12	P.R.22, P.Y.74	P.Y.74 (or P.Y.65)	P.R.22, P.Y.74 (or P.Y.65)
Mix 13	P.R.122, P.Y.1	P.Y.1	P.Y.1
Mix 14	**P.R.122**, **P.R.202**, **P.V.19**	*P.R.177*	–
Mix 15	P.R.254, P.Y.3	P.Y.3	P.R.254, P.Y.3
Mix 16	P.R.254, P.Y.74	P.Y.74 (or P.Y.65)	P.R.254, P.Y.74 (or P.Y.65)
Mix 17	**P.V.19**	*P.R.177*	–
Mix 18	P.Y.14, P.V.23	P.Y.14	P.Y.14, P.V.23

Sum	Wrongly identified Missing	4/18 mixes = 22 % Not applicable	0/37 pigments = 0 % 6/37 pigments = 16.7 %

Two different data evaluation approaches were applied: (1) Average mass spectra (AMS): chromatograms of tattoo inks were converted into AMS and compared to an AMS library made of the 36 pigments under consideration by using the NIST MS program. The best match was taken as a possible hit for pigment identification. Percentage of wrong hits was calculated by division of false identifications by number of inks; (2) Fragment comparison: all peaks at levels of ≥0.2 % of the total peak area were compared to the NIST MS library and the spectra and molecular masses of unknown pyrolysis products (Tables S1–S11); the percentage of wrong hits was calculated by division of false identifications by the total number of pigments present in the inks. Wrongly identified pigments are marked in italics, and pigments that could not be identified in either of the methods are marked in bold as “missing”

**Fig. 2:**
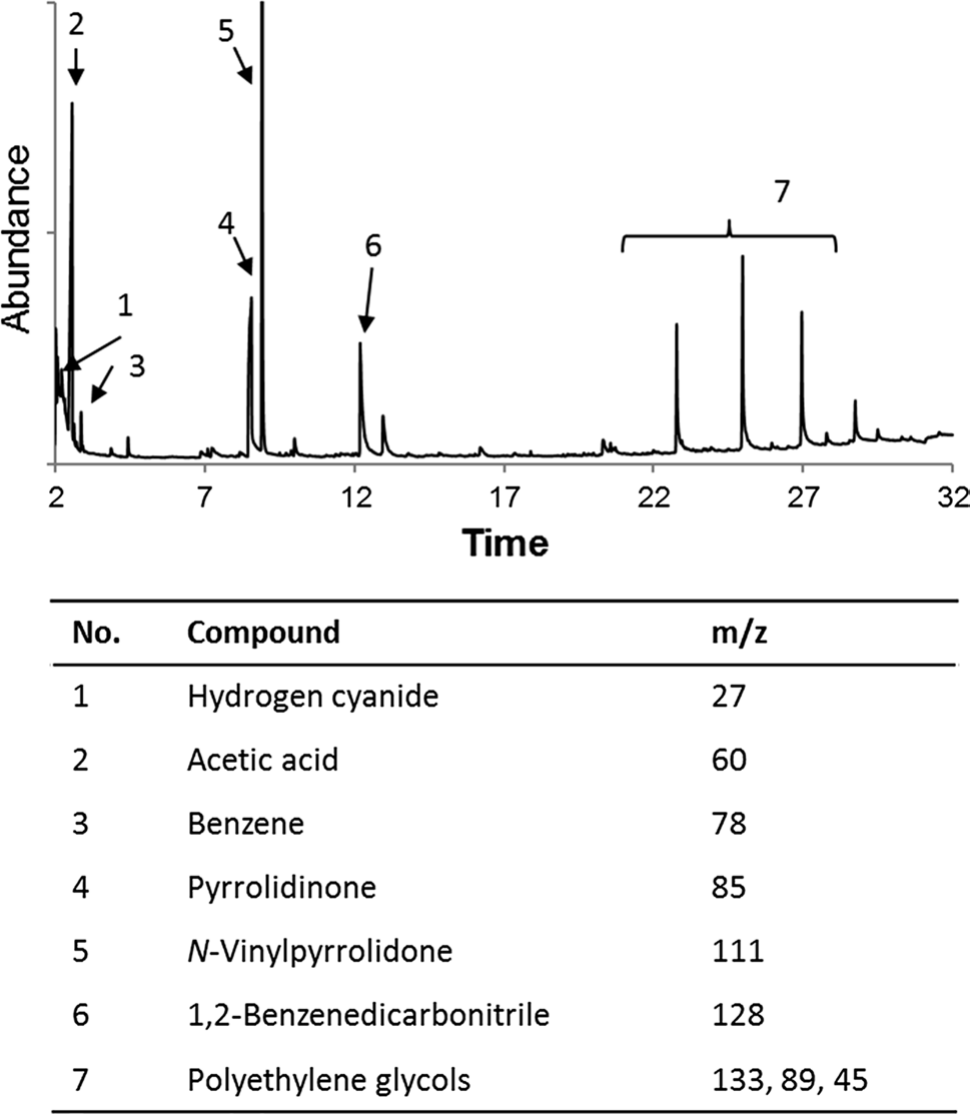
Pyrogram of a blue tattoo ink at 800 °C. Pyrolysis products indicate a tattoo ink formulation containing P.B.15 (hydrogen cyanide, benzene and 1,2-benzenedicarbonitrile), polyvinylpyrrolidone (PVP) (pyrrolidinone and *N*-vinylpyrrolidone) and polyethylene glycol (PEG) derivatives. Most likely, acetic acid was used for pH regulation

Exemplarily, a pyrogram of a blue tattoo ink is displayed in Fig. 2. The product 1,2-benzenedicarbonitrile indicates usage of P.B.15 as main pigment. Also, PVP and PEG have been used, which is verified by the occurrence of pyrrolidinone, *N*-vinylpyrrolidone and various polyethylene glycol derivatives, respectively. Apart from these rather rare inks made of a short ingredient list, combinations of more than one organic pigment are frequently used on the market. A greater variety and higher amounts of components in the inks make pyrograms more complex and identification of ingredients only achievable for experienced personnel. Therefore, we compared two different data evaluation approaches for an easier and faster pyrogram interpretation.

In the first approach, pyrolysis products were manually compared with the fragments compiled in Tables S1–11 (see “Materials and methods” section). Depending on the pigment, 1–3 fragments were required to emerge in the respective pyrogram to sufficiently clarify the identity of the pigment. In total, 80 % of all declared pigments could be identified using this approach (Table 2). As already discussed, tattoo inks are often labeled incorrectly, thereby leaving the possibility that the analysis would be in better agreement with the true composition than the product declaration. In self-made mixtures, the pyrolysis approach did not result in false identification, yet all quinacridone pigments have been missed.

The second approach for pigment identification was a modified version of the method published by Yang et al. (2014). They used a statistical comparison of average mass spectra (AMS) of vehicle top-coatings to describe hierarchical cluster similarity with reference samples of different manufacturers. Unfortunately, this kind of data processing would be only suitable to identify a “brand” rather than single components of the inks. We therefore modified this data evaluation approach using AMS to create a mass spectral library of the 36 pigments pyrolyzed in our study. The AMS of unknown samples were then compared to the AMS library using the NIST MS 2.0 program. High abundant masses from column bleed or other column noises were excluded and masses in the range of 30–400 Da were included in the search. The highest match between a certain tattoo ink and the AMS library was taken as pigment hit. By that, we were able to identify the most abundant pigments in a few seconds in 92.9 % of all tattoo inks and around 80 % in self-made mixtures (Table 2). Hence, this method would facilitate fast and easy screenings, which then can be manually re-assessed using the evaluation approach explained above.

Identification of some pigments in commercially available inks, namely the diazo pigments P.O.13 (ink no. 9–10) and P.Y.14 (ink no. 22–23), the polycyclic P.O.73 (ink no. 12–14) and the quinacridone P.R.202 (ink no. 6 and 14) failed in either of the two evaluation approaches described above (Table 2). Since P.Y.14 and P.O.73 were successfully identified in self-prepared mixtures, their concentrations in the inks were probably too low or they were not present at all. However, the identification of quinacridones and P.O.43 also remained unsuccessful in self-suspended pigment mixtures.

## Conclusions

We were able to prove py-GC/MS suitable for the identification of polymers and pigments used in tattoo inks. Main advantages of this method are the absence of any sample purification steps and a relatively high sensitivity in distinguishing different ingredients of multi-component inks. Py-GC/MS is applicable to a wide range of pigments, including phthalocyanines, diketopyrrolopyrrols, quinophthalones, triphendioxazines and, most importantly, azo pigments. However, in some of the commercial inks investigated some of the pigments could not be identified that have been declared on the label of the container. Occasionally pigments are only used in minute amounts to achieve a certain color shade. Hence, their concentrations might have fallen below the detection limit. Additionally, quinacridones could not be sufficiently identified and thus were a cause for false or incomplete identification. Our results are in accordance with the literature and demonstrate the unsuitability of py-GC/MS for identification of quinacridones (Ghelardi et al. 2014; Russell et al. 2011). Similarly, the polycyclic P.O.43, P.R.179 and other similar structures could not be identified in pigment mixtures. Like quinacridones, these pigments miss specific cleavage sites making identification through py-GC/MS rather difficult.

The unfeasibility to identify pigments without characteristic cleavage sites is a limiting factor of the py-GC/MS approach applied. Methods such as LDI-ToF–MS (insoluble pigments) or LC–MS (soluble pigments) may serve as suitable complementary techniques for the identification of pigments in complex mixtures (Hauri 2014). In addition, FT-IR and Raman spectroscopy might help to pinpoint the most abundant pigments, but will be of limitation in mixtures (Poon et al. 2008; Timko et al. 2004).

Currently, the tattoo ink regulation is highly discussed on the European level (Laux et al. 2016). In the past and due to their insolubility in physiological media, pigments were assumed biochemically inert. In the case of the quinacridones P.V.19 and P.R.122 no toxic endpoint was ever reported based on animal studies of the sole and pure pigments (CPMA 2006). Yet, there were two reported cases of allergic reactions toward tattoo inks containing either of the pigments (Gaudron et al. 2015). Copper phthalocyanine was also judged non-toxic by the OECD (1995). Even azo pigments including P.Y.13 and P.Y.74 did neither show organ toxicity nor mutagenicity or carcinogenicity in animals (Ollgard et al. 1998). It is to be noted, however, that routine toxicity testing does not consider the unique intradermal routes of exposure being operative in tattoos. Unlike irritation and allergic reactions that usually occur directly at the side of the tattoo, the epidemiological proof of systemic adverse effects in humans is extremely difficult and biased by many factors and complex influences.

Although toxicity of the pigments as such is generally not expected it still might result from metabolites or cleavage products emerging after UV light exposure (sun bathing) or laser tattoo removal (Cui et al. 2005; Engel et al. 2007; Hauri and Hohl 2015; Schreiver et al. 2015; Serup and Carlsen 2014). We therefore propose that the pyrogram library introduced—comprising decomposition fragments of the most abundant pigments present in tattoo inks to date—might be used as tool to predict the emergence of potentially toxic and carcinogenic compounds under real-life conditions including laser removal of permanent skin paintings (Engel et al. 2010; Schreiver et al. 2015). To this end, all pyrolysis products of each pigment were also reviewed for their carcinogenicity category according to the GHS classification and their acute oral toxicity (LD_50_ value) in rats (see Tables S1–11). Our data demonstrate that genotoxic pAAs can make up to 20 % of the total peak area in the pyrograms of azo pigments (Table S3). Conversely, other carcinogens such as benzene and naphthalene mostly occur in minor amounts representing only about 1–2 % of the total peak areas. Along with cyanides, both kinds of aromatics are also produced upon combustion of any organic materials, e.g., in fires. It is therefore little surprising that these compounds are released in nearly all of the pigments analyzed. However, effective degradation of pigments may only occur upon laser irradiation at temperatures beyond 800 °C (Schreiver et al. 2015; Engel et al. 2010). On the other hand, none of these fragmentation products have ever been reported upon UV and/or visible light exposure of tattoo pigments (Wezel 2013). This provides evidence that phthalocyanines, quinacridones and other very light fast pigments are advantageous regarding its decomposition behaviors when compared to azo pigments and other amine- or amide-containing pigments such as P.V.37.

## Electronic supplementary material

Below is the link to the electronic supplementary material.

Supplementary material 1 (DOCX 1428 kb)
