# Supplementary material for: Identification and hazard prediction of tattoo pigments by means of pyrolysis—gas chromatography/mass spectrometry
Source: Arch Toxicol. 2016 May 21;90:1639–50. doi: 10.1007/s00204-016-1739-2 (PMC4894928; doi:10.1007/s00204-016-1739-2)
Supplement: Supplementary file 1 — Supplementary material 1 (DOCX 1428 kb) [file 204_2016_1739_MOESM1_ESM.docx]

# Supplements

**Table S1** Pyrolysis products of phthalocyanines. Fragments with more than one m/z specified were concluded by mass spectral interpretation. Hazard categories (Cat.) for carcinogenicity are depicted according to GHS (IPA 2016). Cat. 1A: Known to have carcinogenic potential in humans (evidence from human epidemiology); Cat. 1B: Presumed to have carcinogenic potential in humans (evidence from animal studies); Cat. 2: Suspected human carcinogen. Abbreviations: inh. = inhalative; LD_50_ = lethal concentration required to kill 50% of the animals treated; n.a. = not available.


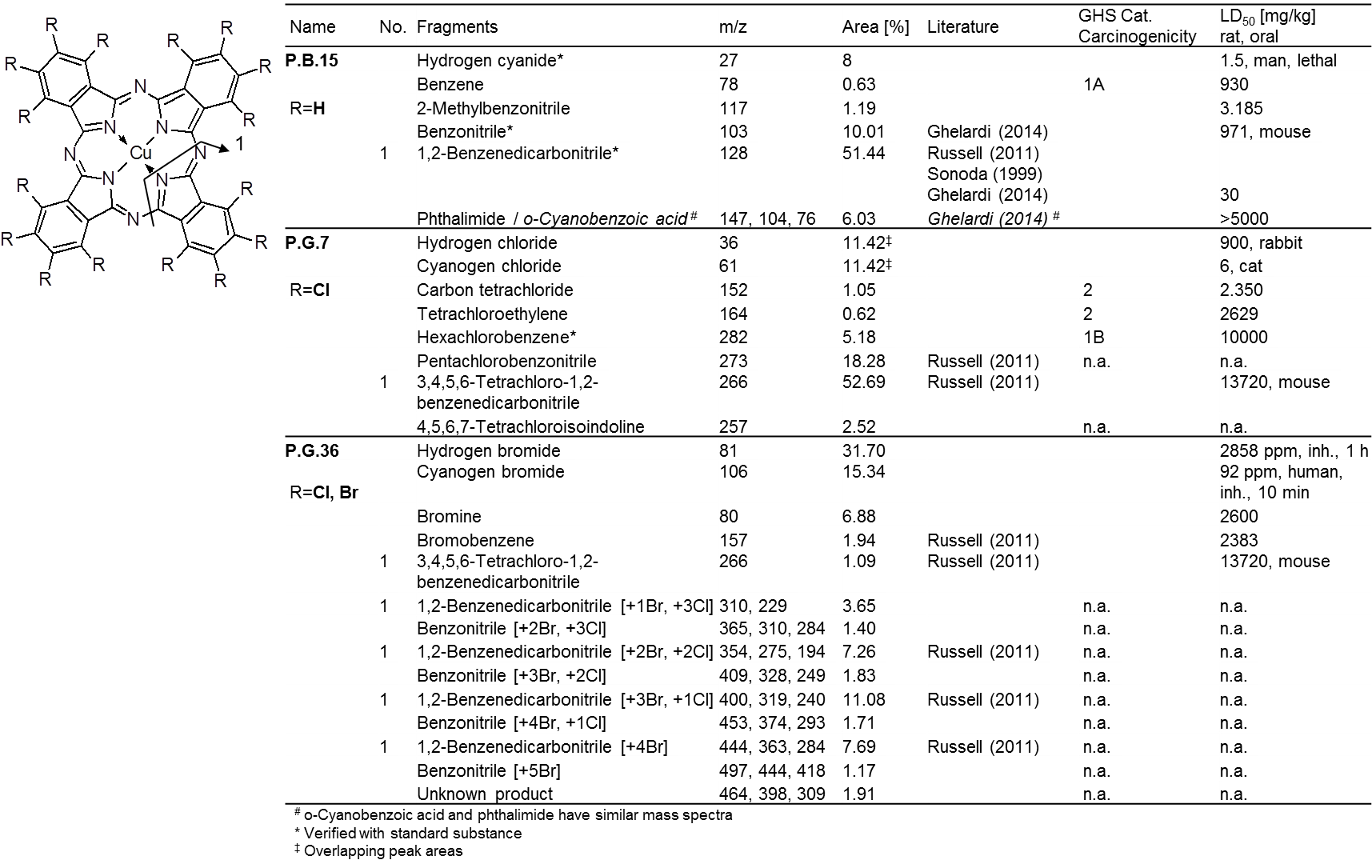


**Table S2** Pyrolysis products of azo/naphthol pigments. Fragments with more than one m/z specified were concluded by mass spectral interpretation. Abbreviations: LD_50_ = lethal concentration required to kill 50% of the animals treated; n.a. = not available.


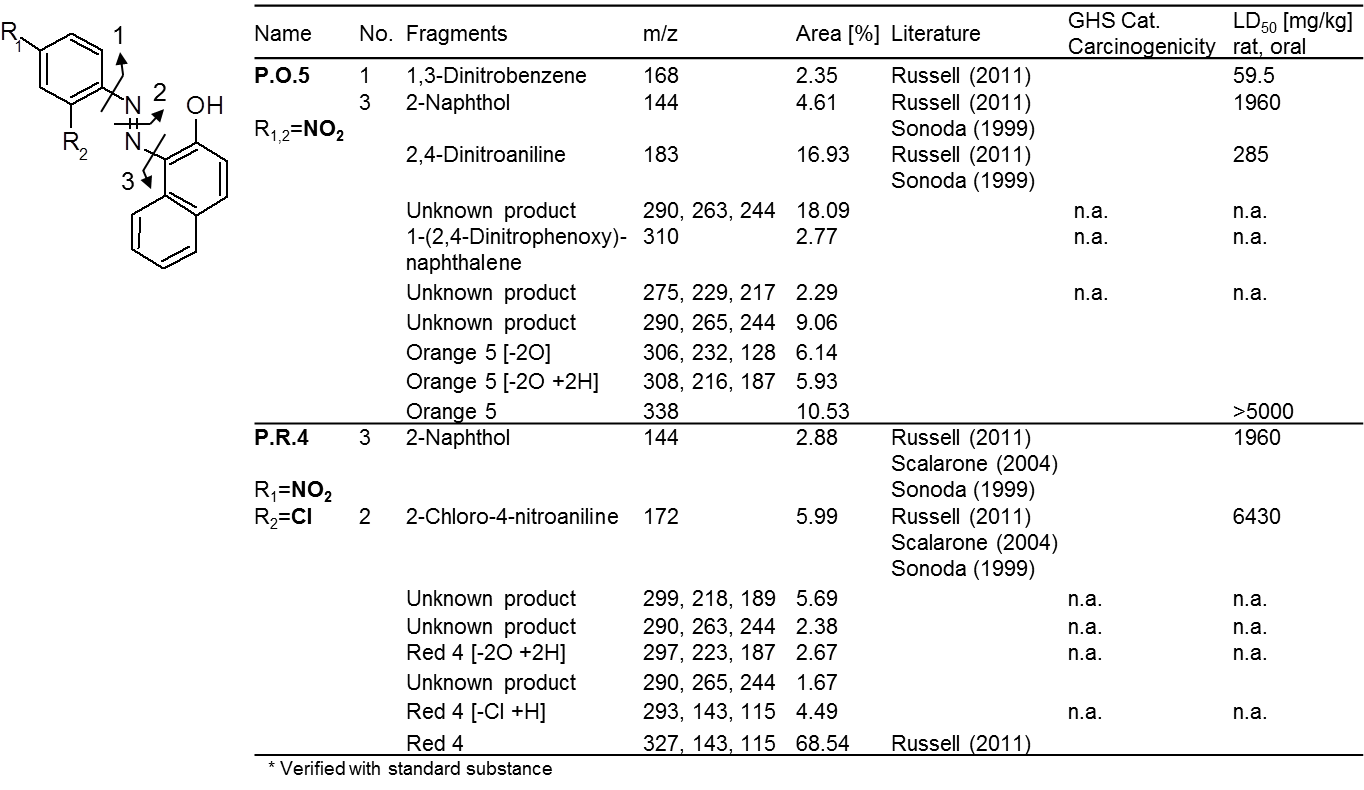


**Table S3** Pyrolysis products of diazo pigments. Fragments with more than one m/z specified were concluded by mass spectral interpretation. Hazard categories (Cat.) for carcinogenicity are depicted according to GHS (IPA 2016). Cat. 1A: Known to have carcinogenic potential in humans (evidence from human epidemiology); Cat. 1B: Presumed to have carcinogenic potential in humans (evidence from animal studies); Cat. 2: Suspected human carcinogen. Abbreviations: iv = intravenous; LD_50_ = lethal concentration required to kill 50% of the animals treated; n.a. = not available.


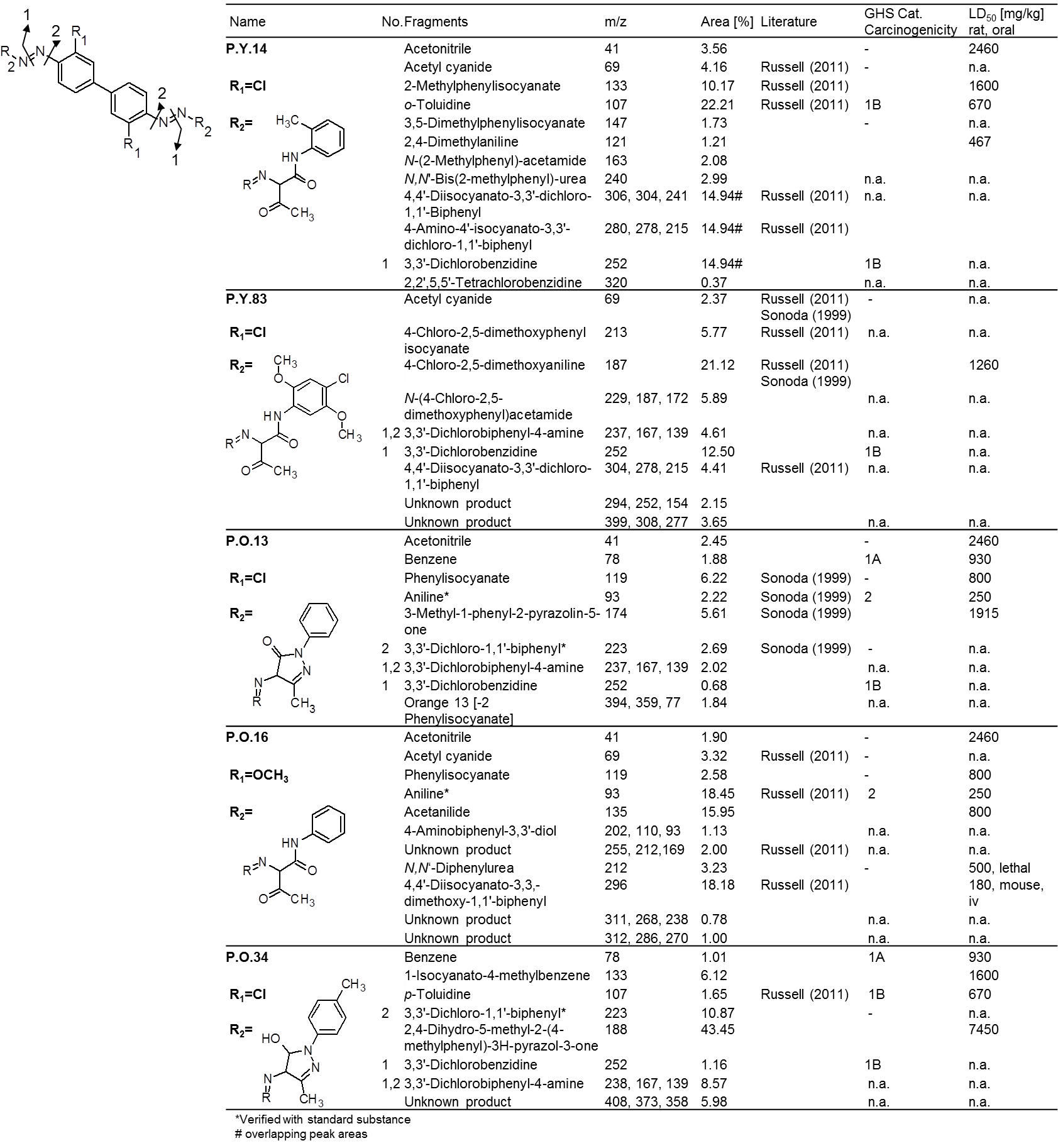


**Table S4** Pyrolysis products of azo/diarylide pigments. Fragments with more than one m/z specified were concluded by mass spectral interpretation. Hazard categories (Cat.) for carcinogenicity are depicted according to GHS (IPA 2016). Cat. 1A: Known to have carcinogenic potential in humans (evidence from human epidemiology); Cat. 1B: Presumed to have carcinogenic potential in humans (evidence from animal studies); Cat. 2: Suspected human carcinogen. Abbreviations: ip = intraperitoneal; iv = intravenous; LD_50_ = lethal concentration required to kill 50% of the animals treated; n.a. = not available.


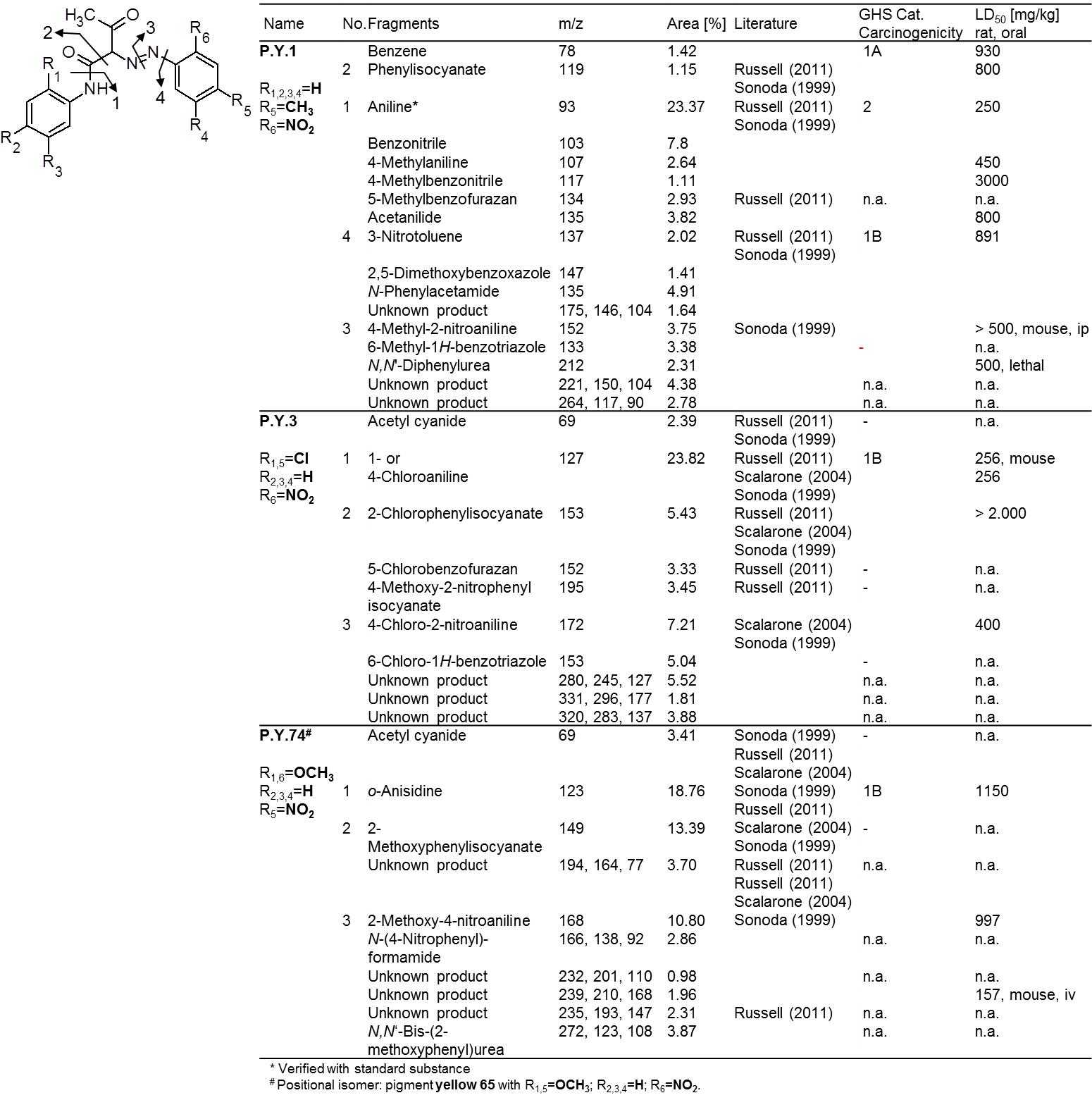


**Table S5** Pyrolysis products of azo pigments. Fragments with more than one m/z specified were concluded by mass spectral interpretation. Hazard categories (Cat.) for carcinogenicity are depicted according to GHS (IPA 2016). Cat. 1A: Known to have carcinogenic potential in humans (evidence from human epidemiology); Cat. 2: Suspected human carcinogen. Abbreviations: LD_50_ = lethal concentration required to kill 50% of the animals treated; n.a. = not available.


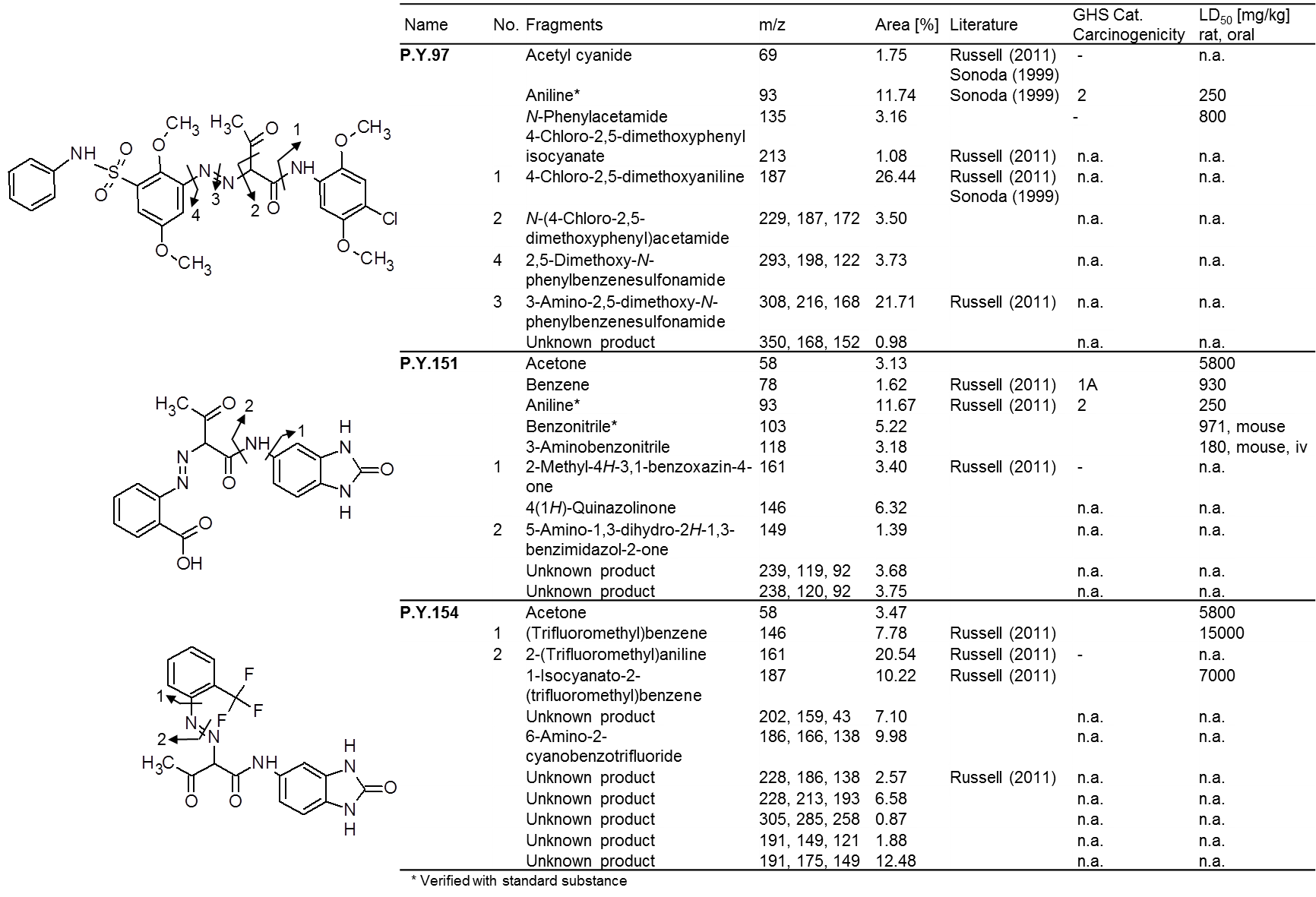


**Table S6** Pyrolysis products of naphthol AS pigments. Fragments with more than one m/z specified were concluded by mass spectral interpretation. Hazard categories (Cat.) for carcinogenicity are depicted according to GHS (IPA 2016). Cat. 1A: Known to have carcinogenic potential in humans (evidence from human epidemiology); Cat. 1B: Presumed to have carcinogenic potential in humans (evidence from animal studies); Cat. 2: Suspected human carcinogen. Abbreviations: iv = intravenous; LD_50_ = lethal concentration required to kill 50% of the animals treated; n.a. = not available.


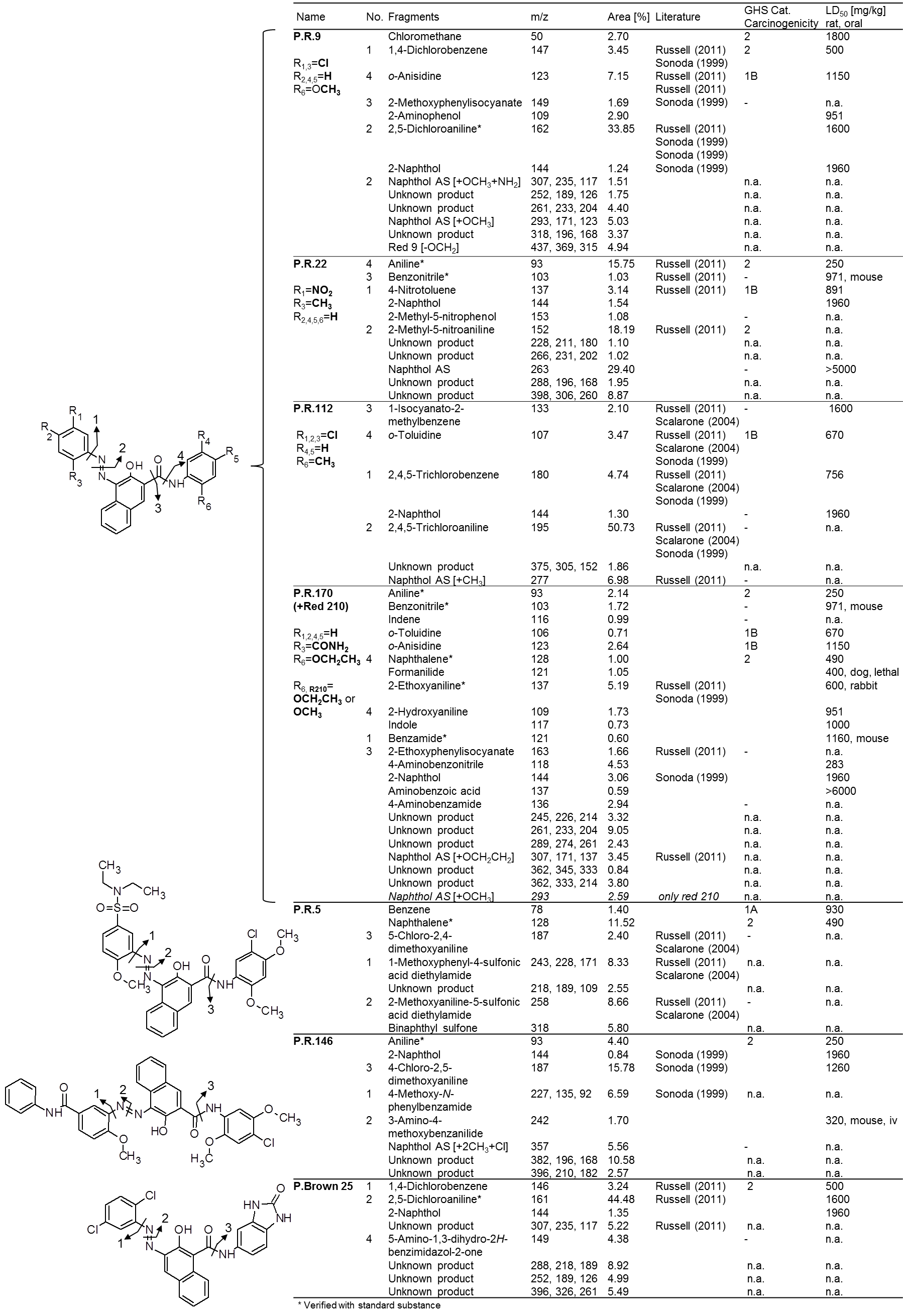


**Table S7** Pyrolysis products of diketopyrrolopyrroles (**pyrrolo[3,2-*b*]pyrrole-diones**). Fragments with more than one m/z specified were concluded by mass spectral interpretation. Hazard categories (Cat.) for carcinogenicity are depicted according to GHS (IPA 2016). Cat. 1A: Known to have carcinogenic potential in humans (evidence from human epidemiology); Cat. 2: Suspected human carcinogen. Abbreviations: ip = intraperitoneal; LD_50_ = lethal concentration required to kill 50% of the animals treated; n.a. = not available.


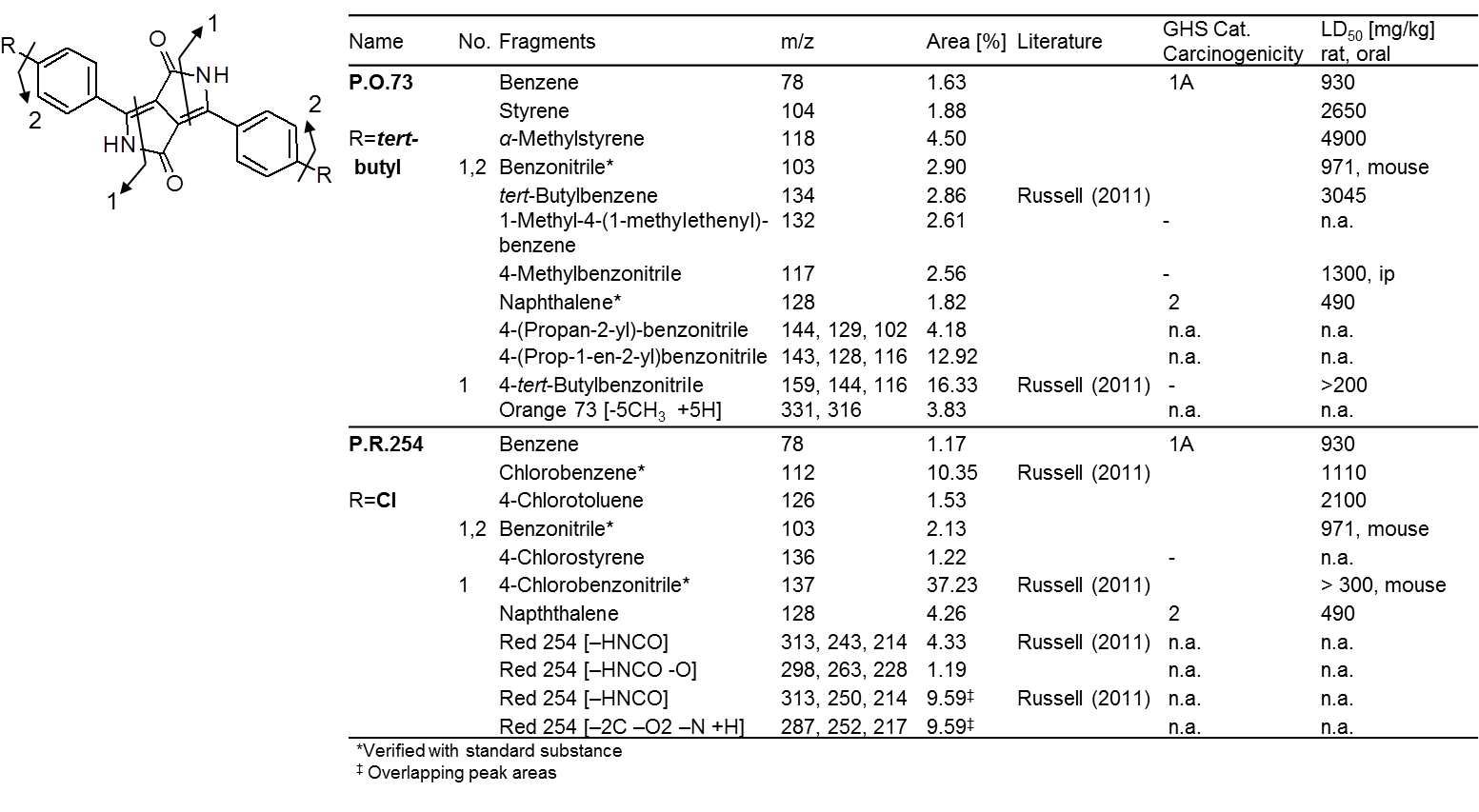


**Table S8** Pyrolysis products of quinophthalones (2-(2-quinolyl)-1,3-indandiones). Fragments with more than one m/z specified were concluded by mass spectral interpretation. Hazard categories (Cat.) for carcinogenicity are depicted according to GHS (IPA 2016). Cat. 1B: Presumed to have carcinogenic potential in humans (evidence from animal studies). Abbreviations: LD_50_ = lethal concentration required to kill 50% of the animals treated; n.a. = not available.


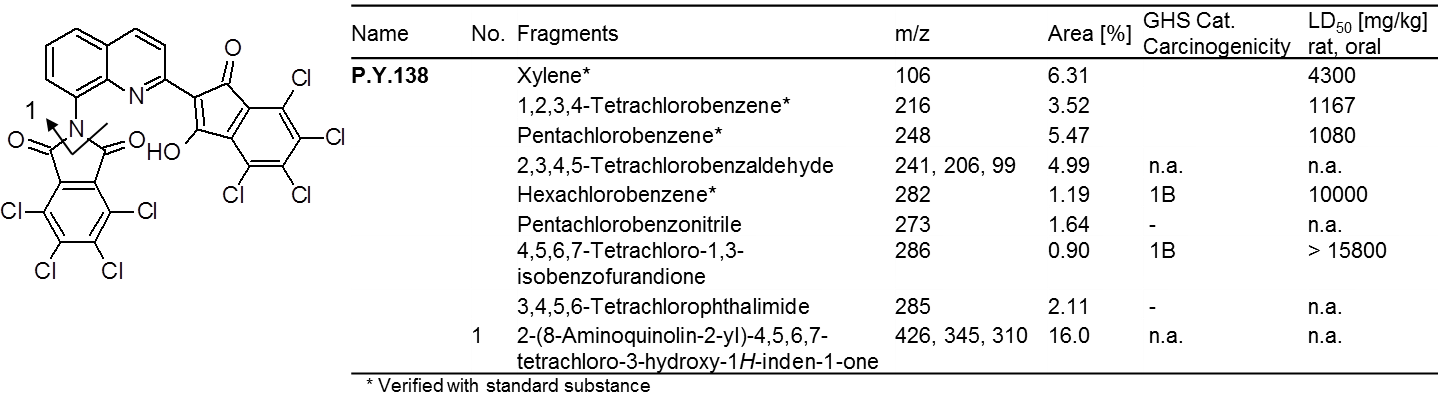


**Table S9** Pyrolysis products of quinacridones (5,12-dihydroquino[2,3-*b*]acridine-7,14-diones). Fragments with more than one m/z specified were concluded by mass spectral interpretation. Hazard categories (Cat.) for carcinogenicity are depicted according to GHS (IPA 2016). Cat. 1A: Known to have carcinogenic potential in humans (evidence from human epidemiology). Abbreviations: LD_50_ = lethal concentration required to kill 50% of the animals treated; n.a. = not available.


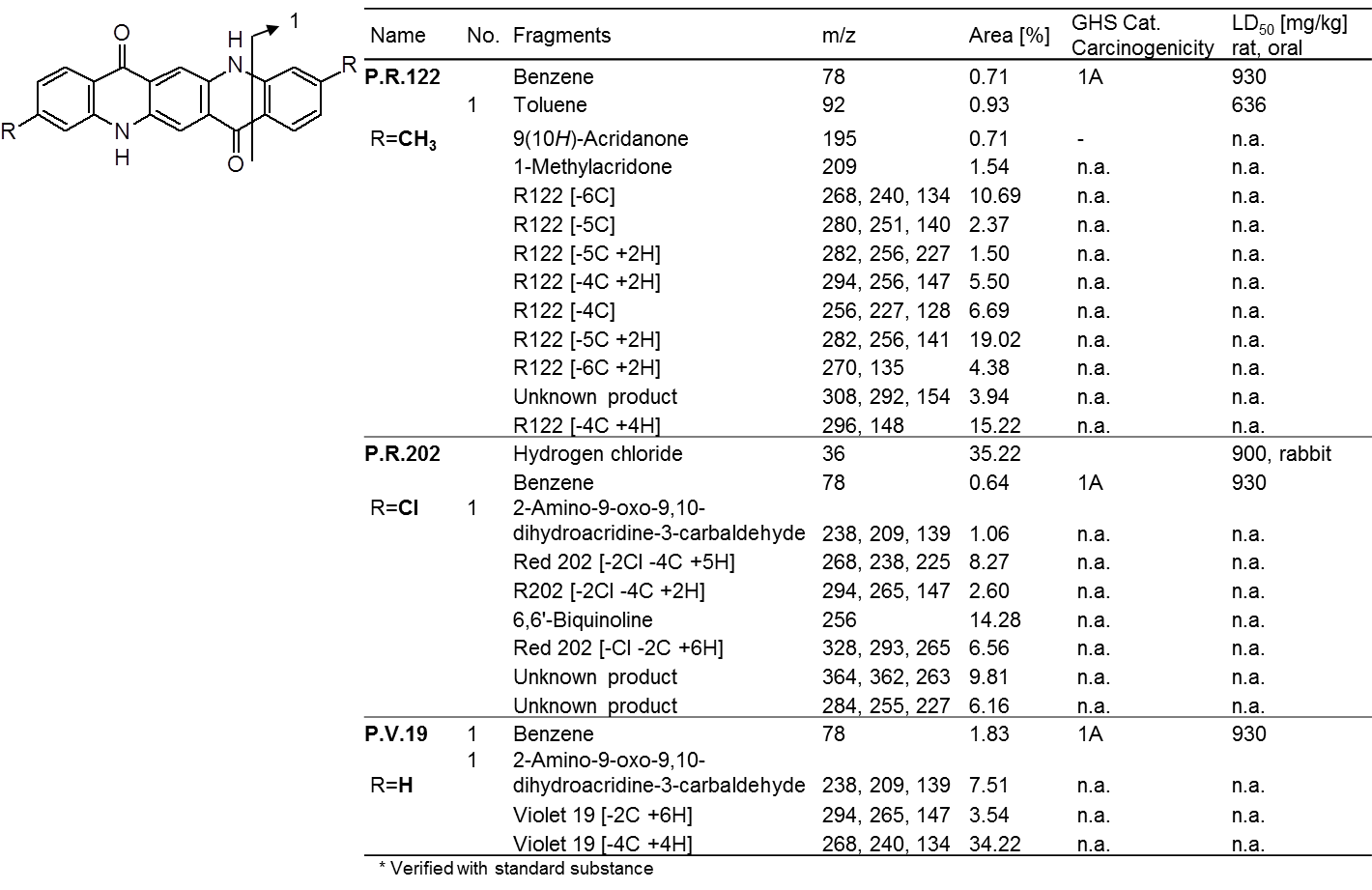


**Table S10** Pyrolysis products of triphendioxazines (‘dioxazines’). Fragments with more than one m/z specified were concluded by mass spectral interpretation. Hazard categories (Cat.) for carcinogenicity are depicted according to GHS (IPA 2016). Cat. 1A: Known to have carcinogenic potential in humans (evidence from human epidemiology); Cat. 2: Suspected human carcinogen. Abbreviations: LD_50_ = lethal concentration required to kill 50% of the animals treated; n.a. = not available.


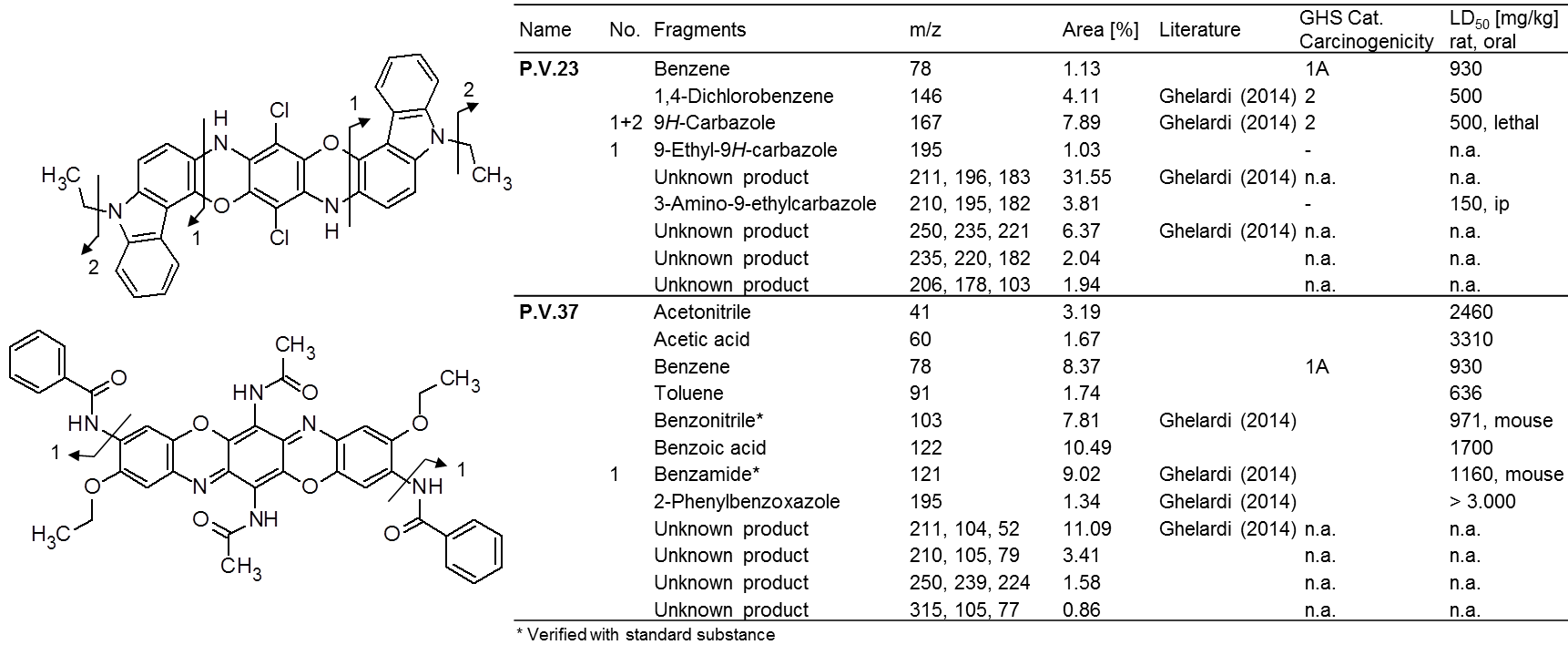


**Table S11** Pyrolysis products of other polycyclic pigments. Fragments with more than one m/z specified were concluded by mass spectral interpretation. Hazard categories (Cat.) for carcinogenicity are depicted according to GHS (IPA 2016). Cat. 1A: Known to have carcinogenic potential in humans (evidence from human epidemiology). Abbreviations: ip = intraperitoneal; LD_50_ = lethal concentration required to kill 50% of the animals treated; n.a. = not available.


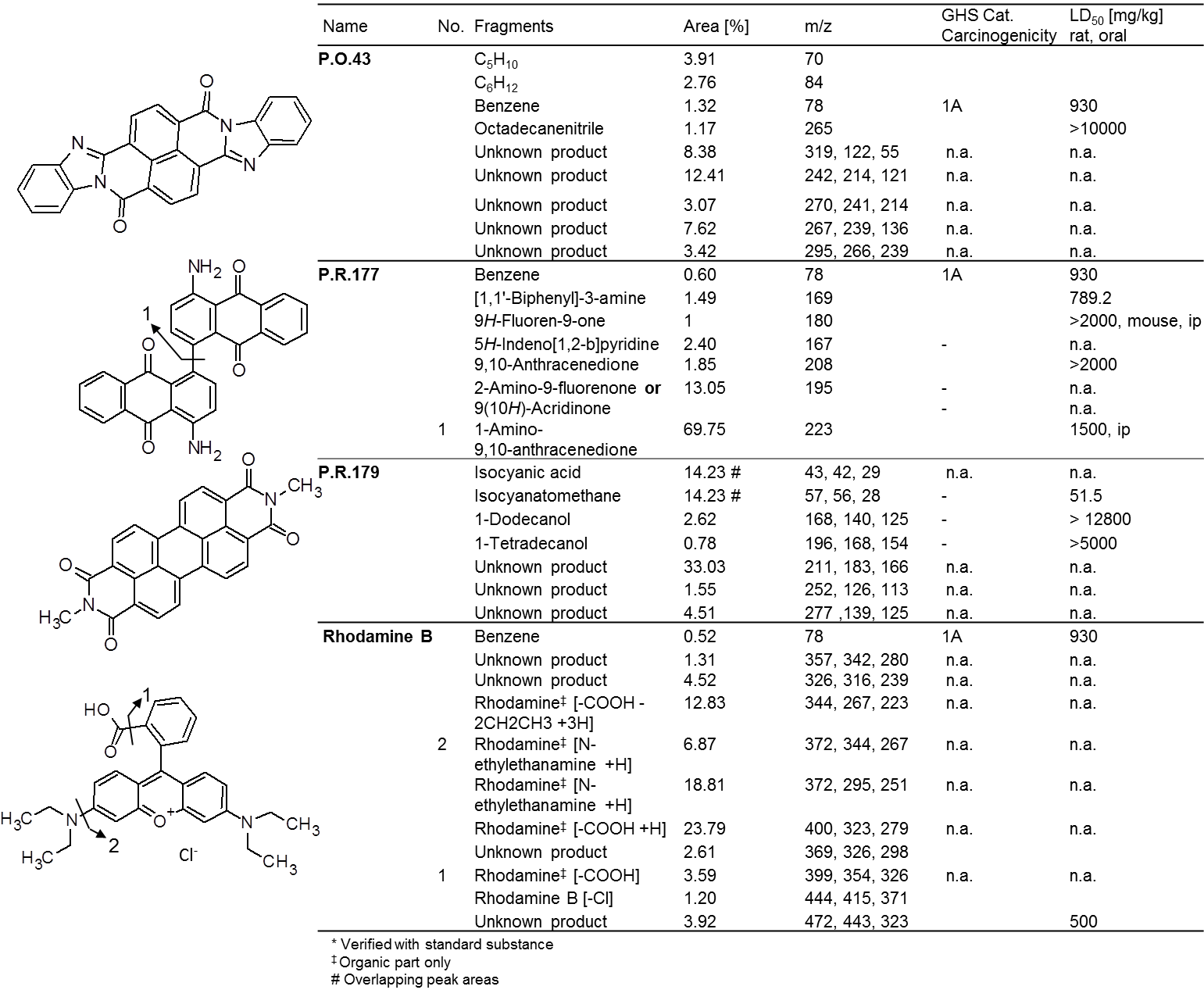


**Table S12** Pigments used to create the pyrogram library.

| No. | Pigment name | Molecular weight | C.I. Number | Trade name (Manufacturer) |
| --- | --- | --- | --- | --- |
| 1 | P.B.15:3 | 576.07 | C.I. 74160 | PV-Echtblau BG-IN (Clariant, Frankfurt am Main, Germany)  Heliogen Blau L 7081 D (BASF, Basel, Switzerland)  Heliogen Blau D 7086 (BASF, Basel, Switzerland)  Puricolor Blue PBL 15:3 (BASF, Basel, Switzerland) |
| 2 | P.Brown 25 | 492.31 | C.I. 12510 | Hostaperm Brown HFR 01 (Clariant, Frankfurt am Main, Germany) |
| 3 | P.G.7 | 1127.19 | C.I. 74260 | PV-Echtgrün GNX (Clariant, Frankfurt am Main, Germany)  Heliogen Grün D 8730 (BASF, Basel, Switzerland)  Puricolor Green PGR7 (BASF, Basel, Switzerland) |
| 4 | P.G.36 | 1660.61 | C.I. 74265 | Heliogen Grün D 9360 (BASF, Basel, Switzerland) |
| 5 | P.O.5 | 338.28 | C.I. 12075 | Hansa Red GG (Clariant, Frankfurt am Main, Germany) |
| 6 | P.O.13 | 623.49 | C.I. 21110 | Graphtol-Orange GPS (Clariant, Frankfurt am Main, Germany)  Irgalite Orange D 2895 (BASF, Basel, Switzerland) |
| 7 | P.O.16 | 620.65 | C.I. 21160 | Irgalite Orange MOR (BASF, Basel, Switzerland) |
| 8 | P.O.34 | 651.54 | C.I. 21115 | Irgalite Orange F2G (BASF, Basel, Switzerland) |
| 9 | P.O.43 | 412.41 | C.I. 71105 | PV Fast Orange GRL (Clariant, Frankfurt am Main, Germany) |
| 10 | P.O.73 | 400.51 | C.I. 561170 | Irgazin Orange DPP RA (Kremer Pigmente, Aichstetten, Germany) |
| 11 | P.R.4 | 327.73 | C.I. 12085 | Irgalite PRR (BASF, Basel, Switzerland) |
| 12 | P.R.5 | 627.11 | C.I. 12490 | 22016 RED (Univar, Billericay, United Kingdom) |
| 13 | P.R.9 | 466.32 | C.I. 12460 | Permanent Red FRLL (Kremer Pigmente, Aichstetten, Germany) |
| 14 | P.R.22 | 426.43 | C.I. 12315 | Naphthanil Red Light RT-531-D (Clariant, Frankfurt am Main, Germany) |
| 15 | P.R.112 | 484.76 | C.I. 12370 | Permanent-Rot FGR (Clariant, Frankfurt am Main, Germany) |
| 16 | P.R.122 | 340.37 | C.I. 73915 | PV-Echtrosa E (Clariant, Frankfurt am Main, Germany)  Cinquasia Pink K 4430 FP (BASF, Basel, Switzerland)  Cinquasia Red L 4100 HD (BASF, Basel, Switzerland) |
| 17 | P.R.146 | 611.04 | C.I. 12485 | Permanent Carmine FBB 02 (Clariant, Frankfurt am Main, Germany) |
| 18 | P.R.170 | 454.48 | C.I. 12475 | Graphtol-Rot F3RK 70-CN09 (Clariant, Frankfurt am Main, Germany) |
| 19 | P.R.177 | 444.43 | C.I. 65300 | Paliogen Red L 4039 (BASF, Basel, Switzerland)  Permanentrot A (Kremer Pigmente, Aichstetten, Germany) |
| 20 | P.R.179 | 418.40 | C.I. 71130 | Paliogen Marron (Kremer Pigmente, Aichstetten, Germany) |
| 21 | P.R.202 | 381.21 | C.I. 73907 | Cinquasia Magenta K 4535 (BASF, Basel, Switzerland) |
| 22 | P.R.210 | 440.45 | C.I. 12477 | Permanent Red F 6RK (Clariant, Frankfurt am Main, Germany) |
| 23 | P.R.254 | 357.19 | C.I. 56110 | PV-Echtrot D3G (Clariant, Frankfurt am Main, Germany)  Irganzin Red K 3840 (BASF, Basel, Switzerland)  Irgazin Red L 3660 HD (BASF, Basel, Switzerland) |
| 24 | P.V.1 | 479.01 | C.I. 45170 | Rhodamine B (Sigma Aldrich, St Louis, MO, USA) |
| 25 | P.V.19 | 312.32 | C.I. 73900 | PV-Echtrosa E3B (Clariant, Frankfurt am Main, Germany) |
| 26 | P.V.23 | 589.47 | C.I. 51319 | PV-Echtviolett RL (Clariant, Frankfurt am Main, Germany) |
| 27 | P.V.37 | 726.73 | C.I. 51345 | Dioxazinviolett (Kremer Pigmente, Aichstetten, Germany) |
| 28 | P.Y.1 | 340.34 | C.I. 11680 | Hansa Yellow G 02 (Clariant, Frankfurt am Main, Germany) |
| 29 | P.Y.3 | 395.20 | C.I. 11710 | Studiogelb (Kremer Pigmente, Aichstetten, Germany) |
| 30 | P.Y.14 | 657.55 | C.I. 21095 | Irgalite Yellow D 1111 (BASF, Basel, Switzerland) |
| 31 | P.Y.74 | 386.36 | C.I. 11741 | Hansa-Brilliantgelb 5GX (Clariant, Frankfurt am Main, Germany)  Irgalite Yellow L 1257 (BASF, Basel, Switzerland) |
| 32 | P.Y.83 | 818.48 | C.I. 21108 | Irgalite Yellow B3RN (BASF, Basel, Switzerland) |
| 33 | P.Y.97 | 591.03 | C.I. 11767 | Novoperm Yellow FGL (Clariant, Frankfurt am Main, Germany) |
| 34 | P.Y.138 | 694.96 | C.I. 56300 | Paliotol Gelb L 0962 HD (BASF, Basel, Switzerland)  Paliotol Gelb D 0960 (BASF, Basel, Switzerland) |
| 35 | P.Y.151 | 381.34 | C.I. 13980 | Permanentgelb hell (Kremer Pigmente, Aichstetten, Germany) |
| 36 | P.Y.154 | 405.33 | C.I. 11781 | Permanentgelb mittel (Kremer Pigmente, Aichstetten, Germany) |
